# Successful Surgical Therapy of a Chronically Infected Thoracic Fistula After Repeated Cardiovascular High‐Risk Procedures

**DOI:** 10.1002/ccr3.71507

**Published:** 2025-11-19

**Authors:** Nikolaus Thierfelder, Eva Riedlinger, Sinan Mert, Benedikt Fuchs, Nicholas Möllhoff, Nikolaus Wachtel, Christian Schneider, Sven Peterß, Riccardo Giunta, Felix Vollbach

**Affiliations:** ^1^ Division of Hand, Plastic and Aesthetic Surgery University Hospital of Munich, LMU Munich Germany; ^2^ Department of Cardiology University Hospital of Munich, LMU Munich Germany; ^3^ Division of Thoracic Surgery University Hospital of Munich, LMU Munich Germany; ^4^ Department of Cardiac Surgery University Hospital of Munich, LMU Munich Germany

**Keywords:** aortic dissection, chronic wound, TAVI, thoracic fistula

## Abstract

Chronic thoracic fistulas after transapical TAVI are rare but serious complications. A 67‐year‐old male with prior aortic dissection and two transapical valve implantations developed a chronically infected fistula due to retained apical closure material. Multidisciplinary surgery with complete removal of infected tissue, staged debridement, vacuum‐assisted closure (VAC) therapy, and targeted antibiotics achieved infection control and successful wound reconstruction using local flaps. The patient recovered uneventfully. Complete foreign material removal and interdisciplinary management are crucial for durable healing in high‐risk posttransapical TAVI patients.

**Summary**

- Chronic thoracic fistulas following transapical TAVI are rare. Complete removal of infected foreign material, staged VAC therapy, and targeted antibiotics within a multidisciplinary approach can achieve infection control and definitive wound closure, even in high‐risk patients with multiple comorbidities.

## 1 Introduction

Treatment of aortic valve disease in patients with prior surgical repair for acute aortic dissection is challenging. Surgical redo procedures carry high perioperative risk, and transcatheter aortic valve implantation (TAVI) is often limited to a transapical approach when vascular access is restricted [1]. While transapical TAVI can achieve outcomes comparable to transfemoral access, it carries increased risks of apical site complications [2]. Chronic thoracic fistulas after transapical TAVI are rare but can occur, particularly in patients with multiple comorbidities. We present a case of a chronically infected thoracic fistula years after transapical TAVI, successfully managed with staged surgical and antimicrobial therapy.

## 2 History of Presentation

**FIGURE 1:**
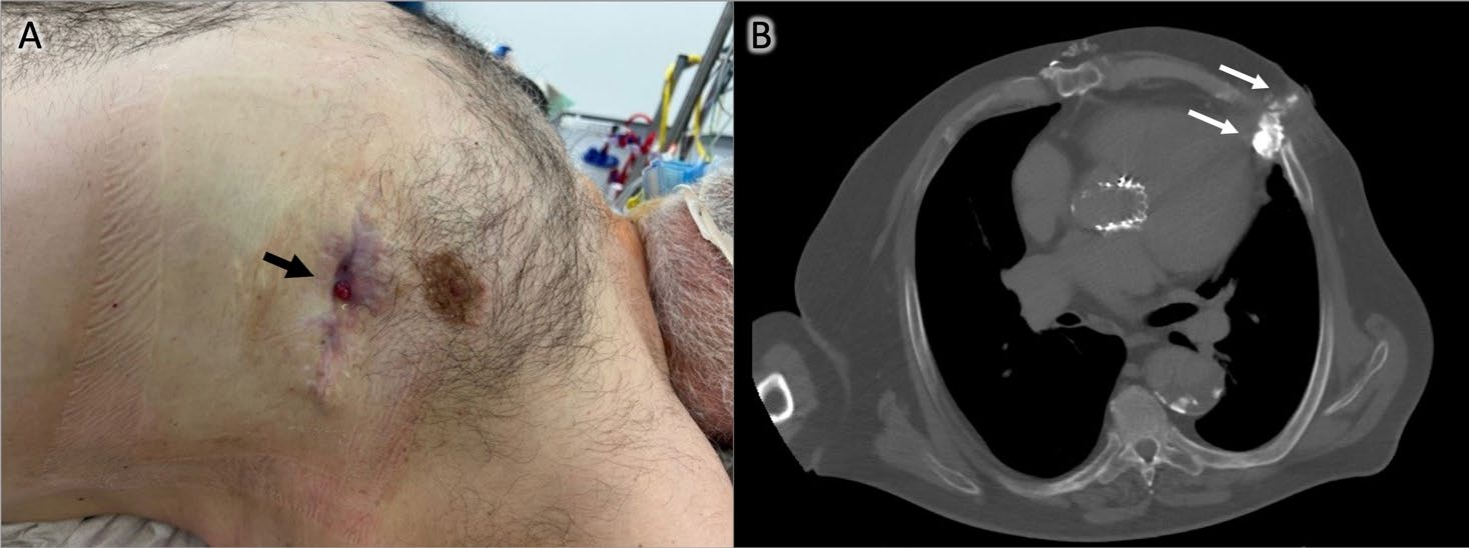
(A) Chronic fistula of the left lateral thorax; (B) CT scan after contrast injection through the chronic wound, showing a fistula reaching the cardiac apex (white arrows).

A 67‐year‐old male presented to our plastic surgery outpatient department with a chronic wound on the left lateral thorax in May 2025. He reported that the fistula had appeared approximately 3 years prior, located in the center of a previous surgical scar. At presentation, the wound (size: 0.5 × 0.5 cm) demonstrated minimal secretion, slight peripheral erythema, and no swelling (see Figure 1A).

The patient was in reduced general condition (weight: 120 kg, BMI: 37 kg/m^2^). Relevant comorbidities included COPD (GOLD stage III, intermittent oxygen therapy), dilated cardiomyopathy (EF 30%), stage III renal failure and type 2 diabetes mellitus.

## 3 Past Medical History

The patient with a complex cardiac history initially presented in an external hospital (September 2014) with an acute Stanford type A aortic dissection, requiring surgical supracoronary replacement of the ascending aorta. The aortic dissection extended from the aortic root into the iliac arteries, with proximal involvement of the brachiocephalic trunk. In April 2015, he underwent early revision surgery with transapical transcatheter aortic valve implantation (TAVI) using a 27 mm JenaValve prosthesis due to symptomatic (NYHA class III) aortic insufficiency (grade II–III), which was attributed sonographically to restriction of the left aortic valve leaflet. The “APICA XL” (Thoratec Corp., USA) apical closure system was utilized during the procedure.

In March 2019, the patient developed symptomatic (NYHA class III) severe aortic insufficiency secondary to a paravalvular leak, for which a repeat transapical TAVI (“valve‐in‐valve”) with a 29 mm Edwards Sapien 3 valve was performed. A repeated transapical approach had to be taken due to persistent dissection membranes in the brachiocephalic trunk and the descending aorta.

In 2022, the patient developed wound dehiscence at the previous surgical implantation site. This necessitated simple secondary wound closure in February 2023. Due to persistent wound healing issues, the patient underwent debridement, vacuum‐assisted closure (VAC) therapy, and secondary wound closure again in February 2024.

## 4 Investigations

Multiple CT examinations were performed over the period of fistula persistence and prior to definitive surgical management. During the last CT (prior to surgical wound therapy) with contrast injection through the fistula, a transcutaneous and transthoracic fistula measuring 6.7 × 2.6 cm, extending to the pericardial fat, was documented (see Figure 1B).

Upon admission, blood tests revealed slightly elevated inflammatory markers (WBC: 12.3 G/L; CRP: 1.3 mg/dL), stage III renal insufficiency (GFR: 59 mL/min), hyperglycemia (223 mg/dL), and mild thrombocytopenia (120 G/L).

## 5 Management

In multidisciplinary consensus (cardiac, thoracic, and plastic surgery teams), a staged approach was planned, including resection, debridement, VAC therapy, and thoracic wall reconstruction.

**FIGURE 2:**
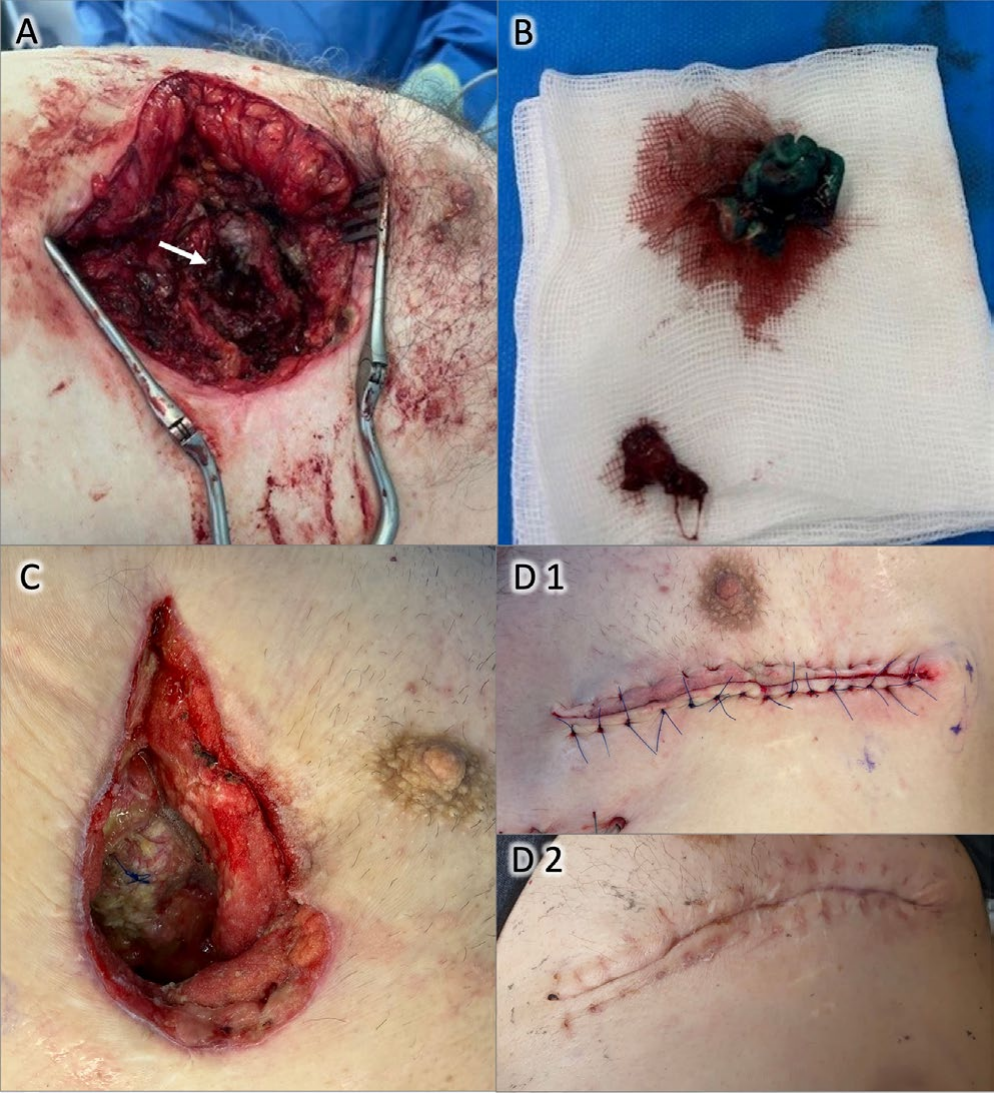
(A) Left mini‐thoracotomy exposing old suture material and reinforcement felt from transapical TAVI (white arrow); (B) Chronically infected suture material and felt after resection; (C) Wound after repeated debridement and VAC therapy; (D1) Closed wound after surgery; (D2) Wound on postoperative day 21 after suture removal.

The fistula was marked with methylene blue, and a lateral mini‐thoracotomy was performed and the corresponding rip was resected. The whole resectate containing the fistula and the surrounding tissue with the rip resectate adhered to the pericardium. A surrounding pericardiotomy was performed to mobilize the preparation and it demonstrated a strong adherence to the apex of the heart. After careful preparation along the scarred tissue an old apical reinforcement of the cardiotomy appeared and sutures were identified (see Figure 2A,B), with surrounding chronically infected tissue. After foreign material removal and debridement, VAC therapy was initiated.

Sequential debridements and VAC exchanges were performed on days 4, 11, and 18. Microbiological analysis identified colonization—especially of the removed foreign material—with *Proteus mirabilis* and *Corynebacterium striatum* . According to the resistogram antibiotic therapy with piperacillin/tazobactam and linezolid was administered. Finally, the defect closure and thorax wall reconstruction were performed on day 26. Intraoperatively, the wound showed good granulation and no signs of persistent infection (see Figure 2C).

Wound was closed in a multilayered approach, beginning with a pectoral muscle reconstruction and tissue mobilization for a local bilateral advancement flap. Deep and superficial subcutaneous sutures and a final skin closure in donati‐backstitch technique (see Figure 2D). For wound protection a supradermal vacuum system was applied for 4 days.

## 6 Outcome and Follow‐Up

The postoperative course was uneventful, with no signs of recurrent infection. Drains were removed in a timely manner, and the patient was discharged on postoperative day 4. Wound healing progressed without complications, with suture removal on postoperative day 21.

## 7 Discussion

Treatment of aortic valve disease is particularly challenging in patients with a history of surgical repair for acute aortic dissection. On the one hand, surgical redo procedures are associated with a high (peri‐) operative risk [3] and technically demanding cannulation for cardiopulmonary bypass. On the other hand, TAVI is often limited to a transapical approach [1], as a persistent dissection of the abdominal aorta or iliac arteries may preclude transfemoral access. However, available evidence shows that transapical TAVI can achieve comparable success rates at increased rates of apical access site complications and bleeding compared to transfemoral access, emphasizing the importance of careful patient selection and postprocedural surveillance [4]. The incidence of paravalvular leak (PVL) remains clinically significant for TAVI [5], with moderate to severe PVL linked to increased long‐term mortality and rehospitalization [6]. Different techniques for PVL treatment are described, whereas valve‐in‐valve is a suitable and effective option [7]. In case of vascular access restriction, as with our patient (aortic dissection), this redo procedure can be performed transapically.

However, repeat transapical interventions increase the complexity of wound care and may predispose to chronic infections due to persistent foreign material, impaired vascularization, and bacterial colonization. Infected intracorporeal material may remain subclinical for many years but can eventually lead to transdermal fistula formation, as observed in our patient. Chronic thoracic fistulas following transapical TAVI are rare but challenging complications [2, 8], particularly in patients with multiple comorbidities such as obesity, COPD, diabetes mellitus, and chronic kidney disease. Management of chronic infected thoracic fistulas post‐TAVI should involve a multidisciplinary approach combining cardiothoracic and plastic surgery as well as infectious disease expertise [9]. Complete excision of infected foreign material is essential, as residual suture or reinforcement materials may perpetuate infection. Vacuum‐assisted closure (VAC) therapy has demonstrated efficacy in enhancing granulation tissue, reducing bacterial load, and controlling exudate in complex thoracic wounds. Recent guidelines and meta‐analyses affirm the role of VAC as a bridge to definitive closure, enabling infection control before (flap‐based) reconstruction [10].

Our case illustrates the successful application of sequential debridement, targeted antibiotic therapy, staged VAC application, and definitive thoracic wall reconstruction using local flaps, ultimately leading to complete wound closure without residual long‐term infection in a high‐risk multimorbid patient. This approach underscores the feasibility of managing complex thoracic wound complications posttransapical TAVI, even in the presence of significant comorbidities, when structured within an interdisciplinary high‐risk protocol.

## 8 Conclusion

This case highlights the rare but significant complication of chronic thoracic fistula formation following transapical TAVI, demonstrating the necessity of complete foreign material removal, effective infection control, and staged wound management using VAC therapy in a high‐risk patient population. Interdisciplinary planning and meticulous surgical execution are essential for successful outcomes in these complex scenarios.

## Author Contributions

**Nikolaus Thierfelder:** conceptualization, data curation, formal analysis, project administration, writing – original draft. **Eva Riedlinger:** formal analysis, writing – review and editing. **Sinan Mert:** data curation, formal analysis, writing – review and editing. **Benedikt Fuchs:** formal analysis, methodology, validation, writing – review and editing. **Nicholas Möllhoff:** formal analysis, methodology, writing – review and editing. **Nikolaus Wachtel:** investigation, methodology, writing – review and editing. **Christian Schneider:** investigation, methodology, writing – review and editing. **Sven Peterß:** data curation, investigation, methodology, writing – review and editing. **Riccardo Giunta:** investigation, project administration, resources. **Felix Vollbach:** data curation, investigation, methodology, writing – review and editing.

## Ethics Statement

The authors have nothing to report.

## Consent

A written informed consent was obtained from the patient.

## Conflicts of Interest

The authors declare no conflicts of interest.

## Data Availability

The data that support the findings of this study are available from the corresponding author, Nikolaus Thierfelder, upon reasonable request.

## References

[1] D. Useini , B. Beluli , H. Christ , et al., “Transapical Transcatheter Aortic Valve Implantation in Patients With Aortic Diseases,” European Journal of Cardio‐Thoracic Surgery 59, no. 6 (2021): 1174–1181. 33709139 10.1093/ejcts/ezab050

[2] K. Narala , S. Banga , S. Gayam , and S. Mungee , “Cutaneo‐Pericardial Fistula After Transapical Approach for Transcatheter Aortic Valve Replacement,” JACC. Cardiovascular Interventions 9, no. 7 (2016): 747–749. 26952911 10.1016/j.jcin.2015.12.020

[3] A. M. Carroll , M. J. Kirsch , M. C. FH , et al., “A Multicenter Analysis of Aortic Root Replacement: Non‐Native Chest Increases Risk of Postoperative Mortality,” Journal of Thoracic and Cardiovascular Surgery 170 (2024): 986–993.e2. 39581307 10.1016/j.jtcvs.2024.11.018

[4] A. Ghatak , C. Bavishi , R. N. Cardoso , et al., “Complications and Mortality in Patients Undergoing Transcatheter Aortic Valve Replacement With Edwards SAPIEN & SAPIEN XT Valves: A Meta‐Analysis of World‐Wide Studies and Registries Comparing the Transapical and Transfemoral Accesses,” Journal of Interventional Cardiology 28, no. 3 (2015): 266–278. 25991422 10.1111/joic.12201

[5] S. Bhushan , X. Huang , Y. Li , et al., “Paravalvular Leak After Transcatheter Aortic Valve Implantation Its Incidence, Diagnosis, Clinical Implications, Prevention, Management, and Future Perspectives: A Review Article,” Current Problems in Cardiology 47, no. 10 (2022): 100957. 34364915 10.1016/j.cpcardiol.2021.100957

[6] H. Takagi and T. Umemoto , “Impact of Paravalvular Aortic Regurgitation After Transcatheter Aortic Valve Implantation on Survival,” International Journal of Cardiology 221 (2016): 46–51. 27400296 10.1016/j.ijcard.2016.07.006

[7] T. Nagasaka , V. Patel , O. Koren , et al., “TAVR‐In‐TAVR With a Balloon‐Expandable Valve for Paravalvular Leak,” Frontiers in Cardiovascular Medicine 11 (2024): 1374078. 38566964 10.3389/fcvm.2024.1374078 PMC10985156

[8] M. Scheid , C. Grothusen , G. Lutter , and R. Petzina , “Cutaneo‐Pericardial Fistula After Transapical Aortic Valve Implantation,” Interactive Cardiovascular and Thoracic Surgery 16, no. 4 (2013): 558–559. 23248166 10.1093/icvts/ivs511 PMC3598026

[9] R. Baillot , É. Fréchette , D. Cloutier , et al., “Surgical Site Infections Following Transcatheter Apical Aortic Valve Implantation: Incidence and Management,” Journal of Cardiothoracic Surgery 7 (2012): 122. 23148583 10.1186/1749-8090-7-122 PMC3541989

[10] Expert Working Group , “Vacuum Assisted Closure: Recommendations for Use. A Consensus Document,” International Wound Journal 5, no. Suppl 4 (2008): 3–19. 10.1111/j.1742-481X.2008.00537.x PMC7951297 18713128

